# Unique Profile of Driver Gene Mutations in Patients With Non-Small-Cell Lung Cancer in Qujing City, Yunnan Province, Southwest China

**DOI:** 10.3389/fonc.2021.644895

**Published:** 2021-04-13

**Authors:** Yongchun Zhou, Feng Ge, Yaxi Du, Quan Li, Jingjing Cai, Xin Liu, Yinjin Guo, Zhenghai Shen, Lincan Duan, Zhan Huang, Fei Yao, Changbin Zhu, Hutao Shi, Yunchao Huang

**Affiliations:** ^1^ Molecular Diagnosis Sub Center of Yunnan Cancer Center, Yunnan Cancer Molecular Diagnosis Center, The Third Affiliated Hospital of Kunming Medical University (Yunnan Tumor Hospital), Kunming, China; ^2^ Yunnan Provincial Key Laboratory of Panax notoginseng, Faculty of Life Science and Technology, Kunming University of Science and Technology, Kunming, China; ^3^ Cancer Center Office, The Third Affiliated Hospital of Kunming Medical University (Yunnan Tumor Hospital), Kunming, China; ^4^ Department of Thoracic Surgery II, The Third Affiliated Hospital of Kunming Medical University (Yunnan Tumor Hospital), Kunming, China; ^5^ Department of Medical Affairs, Amoy Diagnostics Co., Ltd., Xiamen, China; ^6^ Imaging Department, Kunming Tongren Hospital, Kunming, China; ^7^ Department of Thoracic Surgery I, The Third Affiliated Hospital of Kunming Medical University (Yunnan Tumor Hospital), Kunming, China

**Keywords:** *ALK*, *EGFR*, *KRAS*, *ROS1*, mutation profile, non-small-cell lung cancer, Qujing

## Abstract

**Objective:**

Qujing City, Yunnan Province, China, has a high incidence of lung cancer and related mortality. The etiology of NSCLC in Qujing area and distribution of associated molecular aberrations has not been fully elucidated. This study aimed to reveal the profile of driver gene mutations in patients with non-small-cell lung cancer (NSCLC) in Qujing and explore their relationships with clinicopathological characteristics.

**Methods:**

In this study, the mutation profiles of NSCLC driver genes, including *EGFR, ALK, ROS1, KRAS, BRAF, RET, MET, HER2, NRAS*, and *PIK3CA*, were investigated in patients with NSCLC from Qujing and compared with those from other regions in Yunnan Province. The associations between molecular mutations and clinicopathological characteristics were further analyzed.

**Results:**

A distinct profile of driver gene mutations was discovered in patients with NSCLC from Qujing. Interestingly, a higher proportion of *EGFR* compound mutations, including G719X + S768I (19.65% vs 3.38%, P < 0.0001) and G719X + L861Q (21.10% vs 2.82%, P < 0.0001), was observed in patients with NSCLC in Qujing compared with patients in non-Qujing area, besides significantly different distributions of *EGFR* (46.01% vs. 51.07%, *P* = 0.0125), *ALK* (3.17% vs. 6.97%, *P* = 0.0012), *ROS1* (0.5% vs. 2.02%, *P* = 0.0113), and *KRAS* (23.02% vs. 7.85%, *P* < 0.0001). Further, *EGFR* compound mutations were more likely associated with the occupation of patients (living/working in rural areas, e.g., farmers). Moreover, *KRAS* G12C was the dominant subtype (51.11% vs 25.00%, *P* = 0.0275) among patients with NSCLC having *KRAS* mutations in Qujing.

**Conclusions:**

Patients with NSCLC in Qujing displayed a unique profile of driver gene mutations, especially a higher prevalence of *EGFR* compound mutations and dominant *KRAS* G12C subtype, in this study, indicating a peculiar etiology of NSCLC in Qujing. Therefore, a different paradigm of therapeutic strategy might need to be considered for patients with NSCLC in Qujing.

## Introduction

Lung cancer has been the most common cancer globally for more than two decades (1). Every year, 1.8 million people are diagnosed with lung cancer and 1.6 million die of the disease (2). In China, lung cancer has the highest mortality and modality. Qujing City, located in Southwest China, is an area with an extremely high incidence of lung cancer, especially in Xuanwei County (3, 4). Previous studies showed several exposures contributing to a higher incidence of non-small-cell lung cancer (NSCLC), including the use of smoky coal in unvented stoves (5), tobacco smoking (6), food contamination (7), and arsenic and radon among tin miners (8) in this area. These external exposures might lead to a distinct profile of genetic alterations contributing to the occurrence and development of NSCLC. Studies with limited sample size indicated that patients with NSCLC in Xuanwei County had lower *EGFR* and *ALK* mutation rates and a higher rate of *KRAS* mutations (9–11). A higher proportion of *EGFR* exons 18 and 20 co-mutations were also reported (12). In recent years, the discovery of lung cancer driver genes has opened the door to individualized treatment of lung cancer and made the molecular typing of lung cancer more refined (13). Therefore, understanding the real-world driver gene mutation characteristics of patients with NSCLC in the Qujing area is of great importance in unrevealing the genetic etiology and optimizing therapeutic regimens for patients in this region. In this study, 2672 patients with NSCLC from Yunnan Province, including 946 patients from Qujing City, were retrospectively examined. The mutation statuses of lung cancer driver genes *EGFR*, *ALK*, *ROS1*, *KRAS*, *BRAF*, *RET*, *MET*, *HER2*, *NRAS*, and *PIK3CA* were detected. Also, the mutational characteristics of these driver genes were analyzed. A specific profile of mutations in these driver genes was revealed in patients from this region, which might lead to the development of more effective targeted therapeutic interventions for this disease.

## Materials and Methods

### Patients

**Figure 1:**
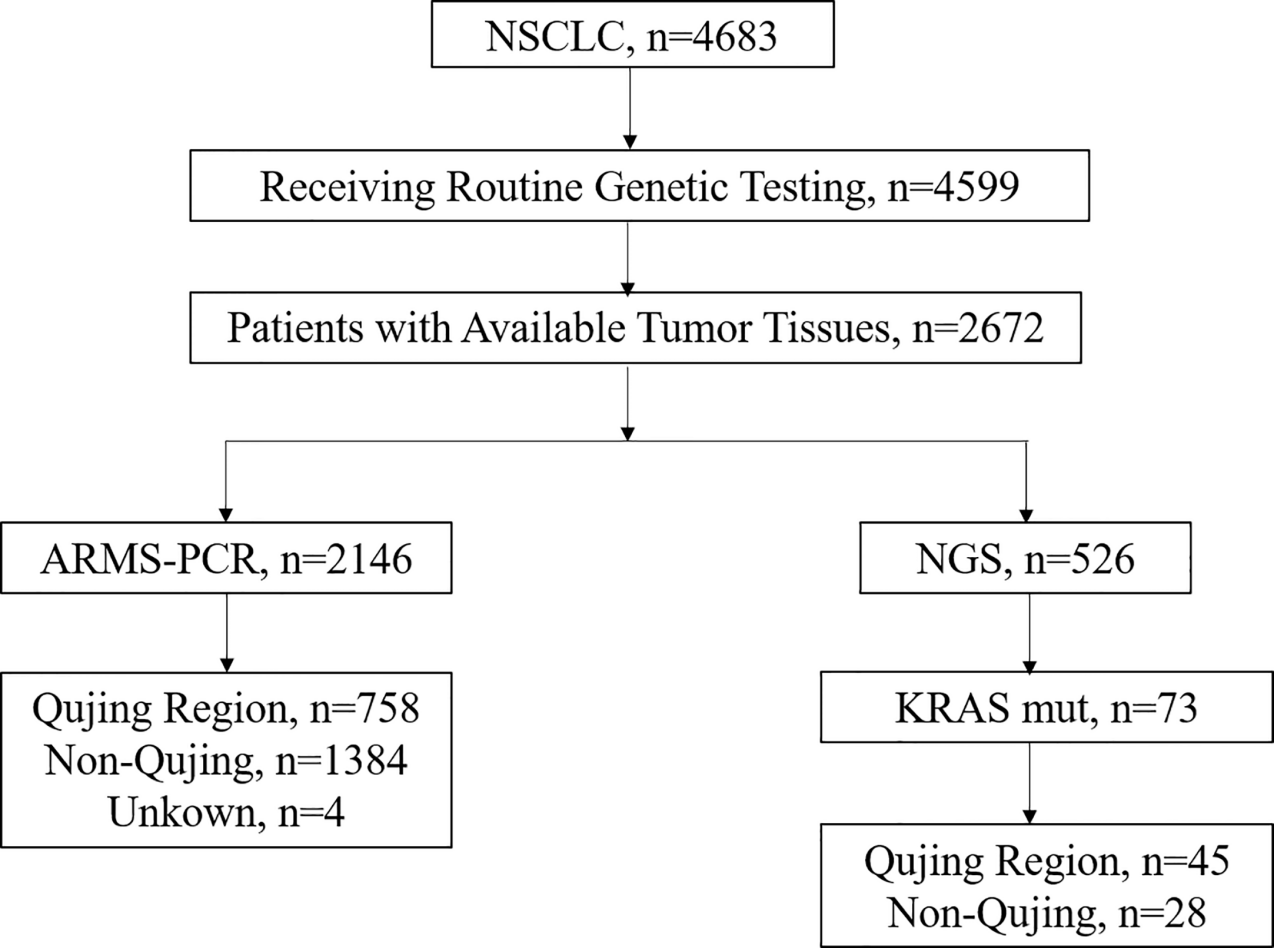
Flowchart of participant selection in this study.

A total of 2672 patients with pathologically diagnosed NSCLC from various regions, including Qujing in Yunnan Province, who visited Yunnan Cancer Hospital between January 2016 and September 2019 were retrospectively recruited (**Figure 1**). This study was conducted with approval from the Institutional Review Board of Yunnan Cancer Hospital. Informed consent was waived because of the retrospective nature of this study, and the de-sensitized clinical data were collected.

### Samples and Mutation Detection

Formalin-fixed paraffin-embedded (FFPE) tumor tissues, or fine‐needle aspiration and/or core needle biopsies, were used to detect mutations in at least one of the following genes, *EGFR*, *ALK*, *ROS1*, *KRAS*, *BRAF*, *RET*, *MET*, *HER2*, *NRAS*, and *PIK3CA*. Genomic DNA and total RNA were extracted from FFPE samples using the AmoyDx FFPE DNA/RNA extraction kit (Amoy Diagnostics, Xiamen, China) following the manufacturer’s protocols. For other types of samples, an AmoyDx Tissue DNA/RNA extraction kit (Amoy Diagnostics) was used. An Amplification Refractory Mutation System Polymerase Chain Reaction (ARMS-PCR) and a Mutation Detection Kit (Amoy Diagnostics) were used to detect the mutations in driver genes (*n* = 2146). The other 526 specimens were captured using commercially available panels and subjected to next-generation sequencing (NGS) following manufacturer’s protocols (**Table 1** and **Supplementary Table 1**).

**Table 1:** Characteristics of patients with NSCLC from Qujing and non-Qujing areas.

Characteristic	All patients (n=2142)	Region		*P-value*
		Qujing (n=758)	Non-Qujing (n=1384)	
**Gender**				
Male	1026 (47.90%)	357 (47.10%)	669 (48.34%)	
Female	1116 (52.10%)	401 (52.90%)	715 (51.66%)	0.3901
**Age**				
Median (range)		53 (17-92)	55 (24-89)	
≤40	109 (5.09%)	42 (5.54%)	67 (4.84%)	
>40	2033 (94.91%)	716 (94.46%)	1317 (95.16%)	0.481
**Histopathology**				
Adenocarcinoma	1978 (92.34%)	726 (95.78%)	1252 (90.46%)	
Squamous carcinoma	157 (7.33%)	32 (4.22%)	125 (9.03%)	<0.001
Unknown (NSCLC)	7 (0.33%)	0	7 (0.51%)	
**Smoking history**				
Yes	672 (31.37%)	251 (33.11%)	421 (30.42%)	
No	1454 (67.88%)	504 (66.49%)	950 (68.64%)	0.2285
Unknown	16 (0.75%)	3 (0.40%)	13 (0.94%)	
**Family history**				
Yes	187 (8.73%)	96 (12.66%)	91 (6.58%)	
No	1954 (91.22%)	661 (87.20%)	1293 (93.42%)	<0.001
Unknown	1 (0.05%)	1 (0.13%)	0	
**Staging**				
I-IIIa	988 (46.13%)	411 (54.22%)	577 (41.69%)	
IIIb-IV	976 (45.56%)	345 (45.51%)	631 (45.59%)	0.0044
Unknown	251 (11.72%)	75 (9.89%)	176 (12.72%)	
**Lesion site**				
Left	769 (35.90%)	229 (30.21%)	540 (39.02%)	
Right	1255 (58.59%)	446 (58.84%)	809 (58.45%)	0.0076
Unknown	48 (2.24%)	13 (1.72%)	35 (2.53%)	
**Occupation**				
Farmer	1005 (46.92%)	460 (60.39%)	545 (39.38%)	
Non-farmer/Unknown	1137 (53.08)	298 (39.31%)	592 (42.77%)	<0.001

### Statistical Analysis

SPSS23.0 (SPSS version 23.0 for Windows, IBM Inc., IL, USA) was used to analyze the relationship between gene mutations and clinicopathological characteristics with the help of *χ* ^2^ test, Fisher’s exact test, or binary logistic regression. The two‐sided significance level was set at *P <*0.05.

## Results

### Clinicopathological Characteristics of Patients With NSCLC in Qujing and Non-Qujing Regions

Among the 2146 patients with NSCLC tested by ARMS-PCR, 758 (35.25%) were from Qujing and 1384 (64.75%) were non-Qujing patients. Regional information was not available for the remaining four patients. The clinicopathological characteristics, including sex, age at diagnosis, smoking history, staging, histopathology, family history, ethnic, lesion site, and metastasis, are listed in **Table 1**. No difference in baseline characteristics was found between these two patient groups. The clinicopathological characteristics of other 526 specimens tested using NGS are shown in **Supplementary Table 2**.

### Mutational Status of Driver Genes in Patients With NSCLC in Qujing

**Figure 2:**
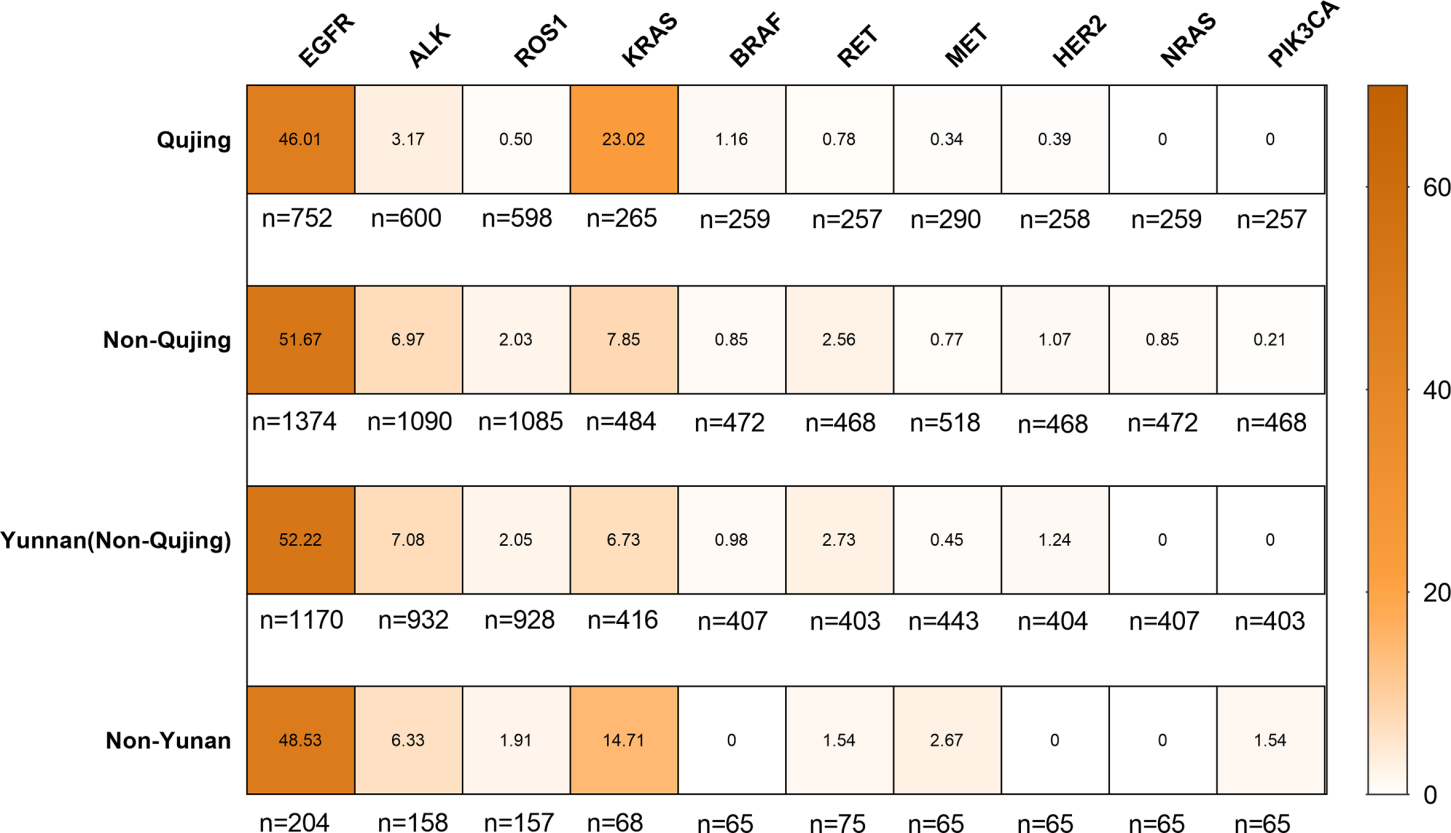
Mutation frequencies of 10 lung cancer driver genes in patients with NSCLC according to the regions in Yunnan Province.

**Figure 3:**
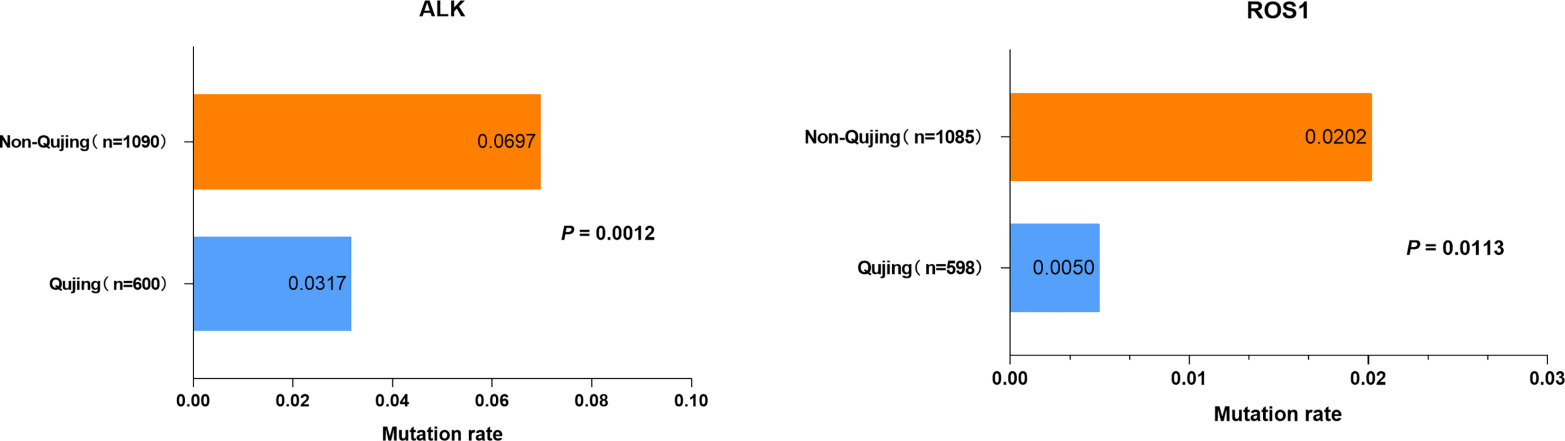
Mutation frequencies of *ALK* and *ROS1* in patients with NSCLC from Qujing and non-Qujing regions.

**Figure 4:**
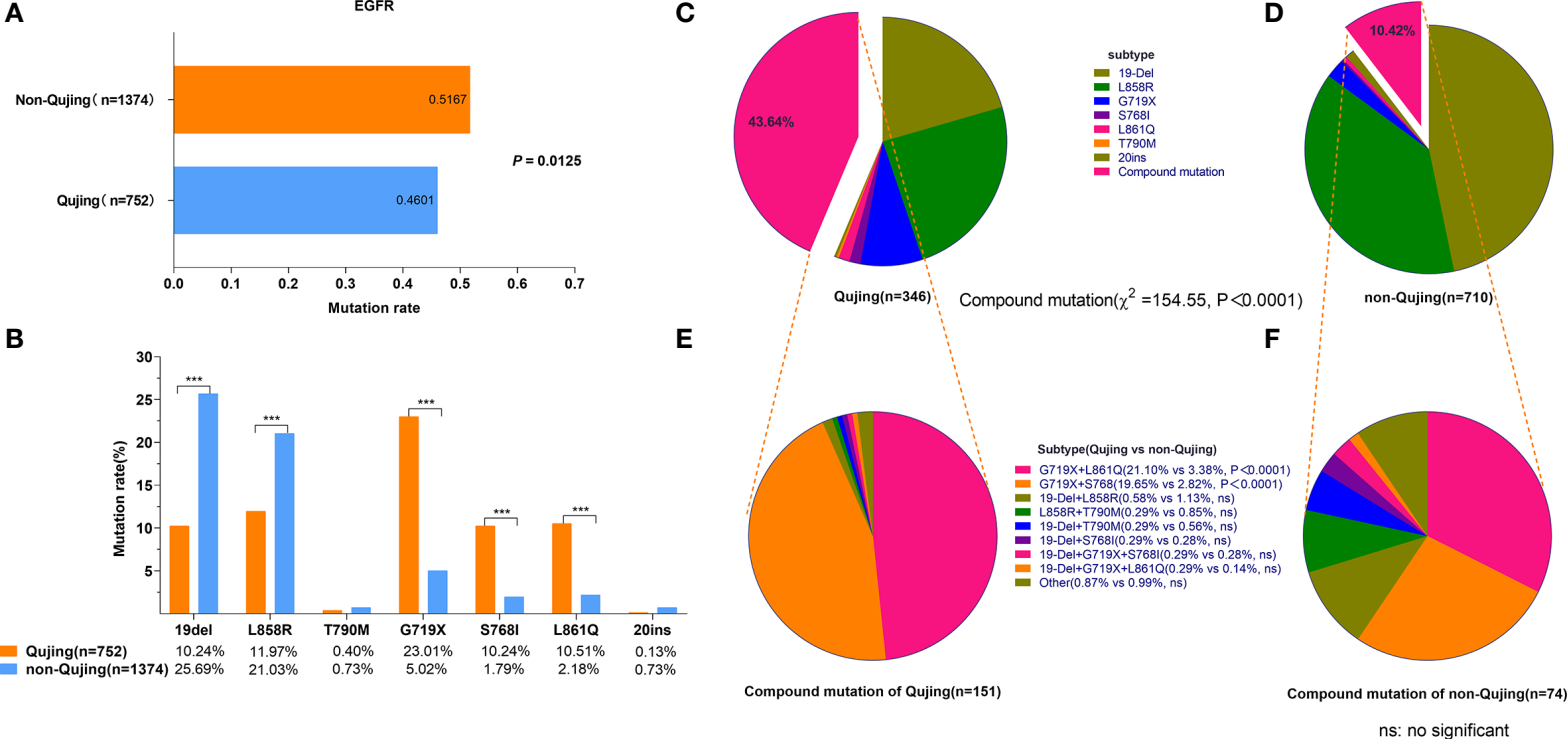
Frequencies of *EGFR* mutations, *EGFR* subtypes, and compound mutations in patients with NSCLC from Qujing and non-Qujing areas. **(A)** the prevalence of *EGFR* mutation in Qujing patients was compared with non-Qujing patients. **(B)** the point mutation frequencies of G719X, S768I, and L861Q were higher in Qujing patients, and the point mutation of 19Del and L858R were lower in Qujing patients. **(C, D)** the distribution of *EGFR* mutation subtypes in Qujing and non-Qujing patients. **(E, F)** the distribution of *EGFR* compound mutation subtypes in Qujing and non-Qujing patients.

**Figure 5:**
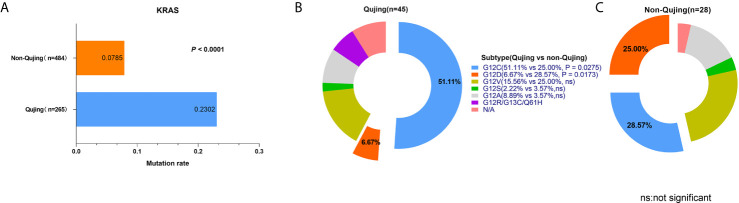
Profile of *KRAS* mutation and subtypes in patients with NSCLC from Qujing and non-Qujing areas. **(A)** the prevalence of *KRAS* mutation in Qujing patients was compared with non-Qujing patients. **(B, C) **the distribution of KRAS mutation subtypes in Qujing and non-Qujing patients.

Among 2142 patients with NSCLC, 1978 were diagnosed with lung adenocarcinoma (92.34%) and 157 had lung squamous carcinoma (7.33%). The landscape of driver mutations in patients with NSCLC from Qujing, non-Qujing, Yunan (non-Qujing), and non-Yunnan regions displayed a region-specific mutational profile (**Figure 2**). Especially, the prevalence of *EGFR* (46.01% vs 51.07%, *P* = 0.0125), *ALK* (3.17%% vs 6.97%%, *P* = 0.0012), and *ROS1* (0.5% vs 2.02%, *P* = 0.0113) was significantly lower in patients from Qujing than in those from non-Qujing regions. On the contrary, the *KRAS* mutation rate was significantly higher in patients with NSCLC in Qujing compared with non-Qujing patients (23.0214% vs 7.85%, *P* < 0.0001) (**Figures 3**, **4A**, and **5A**). Similar results were also obtained in patients with lung adenocarcinoma (**Supplementary Figure 1**). In addition, fewer patients with NSCLC in Qujing had co-mutations of these 10 driver genes compared with those from non-Qujing areas (**Supplementary Figure 2**). Among 526 specimens tested using NGS, the prevalence of *ALK* and *KRAS* mutations in NSCLC patients from Qujing and non-Qujing areas was as similar as the results by ARMS-PCR (**Supplementary Table 3**).

### Relationship Between Clinical Characteristics and *EGFR*, *ALK/ROS1*, and *KRAS* Mutation Statuses in Patients With NSCLC in Qujing

Further, the relationship between *EGFR*, *KRAS*, and *ALK/ROS1* mutation statuses and clinical characteristics in patients with NSCLC in Qujing was analyzed. *EGFR* mutations were more common in female patients with adenocarcinoma, nonsmoker patients with NSCLC (*P* < 0.0001), and patients aged more than 40 years (*P =* 0.0196). *ALK/ROS1* fusions occurred more in patients younger than 40 years old (*P* = 0.0002). However, *KRAS* mutations were more frequent in men (*P* = 0.0066) and smokers (*P* = 0.0084) (**Table 2** and **Supplementary Table 4**).

**Table 2:** Relationship between clinical characteristics and *EGFR, ALK/ROS1*, and *KRAS* mutations in patients with NSCLC from Qujing.

Characteristics	EGFR				ALK/ROS1				KRAS				
	Mut	WT	χ2	*p*	Mut	WT	χ2	*p*	Mut		WT	χ2	*p*
**Gender**													
Male	120	235			13	269			39		90		
Female	226	171	40.34	<0.0001	9	307	1.305	0.2532	22		114	7.381	0.0066
**Age**													
≤40	12	30			5	27			4		7		
>40	334	376	5.446	0.0196	17	549	13.62	0.0002	57		197	1.153	0.2829
**Histopathology**													
AD	343	374			21	554			59		196		
SCC	3	29	18.23	<0.0001	1	22		0.5847	2		8	0.05345	0.8172
**Smoking history**													
Yes	74	176			9	184			26		51		
No	272	227	40.58	<0.0001	12	391	1.091	0.2963	35		152	6.953	0.0084
**Family history**													
Yes	45	50			4	76			10		34		
No	301	335	5.67E-05	0.994	18	500	0.4549	0.5	51		170	0.0025	0.9599
**Ethnic**													
Han	333	339			22	559			60		196		
Non-Han	13	7	1.854	0.1734	0	17		1^*^	1		8		0.6892^*^
*Staging													
I-IIIa	196	213			10	328			40		127		
IIIb-IV	116	153	1.504	0.22	9	189	0.9196	0.3376	18		51	0.1201	0.729
**Lesion site**													
Left	138	158			9	217			26		73		
Right	204	239	0.023	0.8786	13	354	0.07582	0.783	34		129	1.019	0.3128
**Occupation**													
Farmer	214	242			10	341				35	112		
Non farmer/unknown	132	164	0.394	0.5302	12	235	1.652	0.1987		26	92	0.1165	0.7329

AD, adenocarcinoma; SCC, squamous cell carcinoma; *Fisher exact test.

### Distribution of *EGFR* Mutation Subtypes in Patients With NSCLC in Qujing

The mutation frequencies of G719X (23.01% vs 5.02%, *P* < 0.0001), S768I (10.24% vs 1.79%, *P* < 0.0001), and L861Q point mutations (10.51% vs 2.18%, *P* < 0.0001) were significantly higher, while the prevalence of 19Del (10.24% vs 25.69%, *P* < 0.0001) and L858R point mutations (11.97% vs 21.03%, *P* < 0.0001) was significantly lower in patients with NSCLC from Qujing compared with those from non-Qujing areas (**Figure 4B**). In addition, a significantly higher proportion of *EGFR* compound mutations were detected (43.35% vs 10.12%, *P* < 0.0001, for all *EGFR* mutations) (**Figures 4C, D**), and the proportion of *EGFR* G719X + L861Q (21.10% vs 3.38%, *P* < 0.0001) and *EGFR* G719X + S768I (19.65% vs 2.82%, *P* < 0.0001) subtypes was significantly higher in patients with NSCLC from Qujing (**Figures 4E, F**). The multivariate analysis showed that the occupation of patients (living/working in the rural area, e.g., farmers) (odds ratio, 1.923; 95% confidence interval, 1.179–3.137) was independently associated with an increased rate of *EGFR* compound mutations (**Table 3**).

**Table 3:** Multivariate logistic regression analysis of correlations between baseline characteristics and *EGFR* compound mutations in patients with NSCLC from Qujing.

Characteristics	Exp(B)	EXP(B) 95% CI		*P-value*
		upper	lower	
Sex (Male vs Female^*^)	0.986	0.499	1.948	0.969
Age	0.993	0.967	1.020	0.628
Occupation (Farmer vs non-Farmer^*^)	1.923	1.179	3.137	0.009
Race (Han vs non-Han^*^)	1.313	0.368	4.690	0.675
Smoking (Yes vs No^*^)	1.145	0.519	2.527	0.738
Family history (Yes vs No^*^)	1.223	0.622	2.403	0.560
Lesion (Left vs Right^*^)	0.991	0.621	1.581	0.969
Stage (I-IIIa vs IIIb-IV^*^)	0.779	0.484	1.255	0.305
Histopathology (AD vs SCC^*^)	1.502	0.121	18.679	0.752

^*^reference variable.

### Mutational Profile of *KRAS* Subtypes in Patients With NSCLC From Qujing

A total of 73 patients harbored *KRAS* mutations (13.88%) among 526 patients receiving NGS testing, including 45 patients from Qujing and 28 patients from non-Qujing areas. The mutation frequency of *KRAS* G12C was significantly higher in patients with NSCLC from Qujing than in those from non-Qujing areas (51.11% vs 25.00%, *P* = 0.0275). However, the frequency of *KRAS* G12D was significantly lower in patients with NSCLC from Qujing than in those from non-Qujing areas (6.17% vs 28.57%, *P* = 0.0173) (**Figures 5B, C**).

## Discussion

Lung cancer in Qujing City (including Xuanwei County), Yunnan, has four remarkable features: higher incidence, higher mortality, adenocarcinoma as the main histological type, and similar incidence in men and women (5). However, the mutation status of lung cancer driver genes has not been thoroughly investigated among the populations in this region due to the lack of a large-sized study cohort.

Previous studies suggested that lung cancer in the Qujing area had unique epidemiological characteristics due to severe air pollution and the toxicology of indoor coal-fired particles (4, 14–16). This area had more female patients with lung cancer, indicating the presence of strong carcinogens in the body, which had different effects on men and women (17). This study showed that patients with NSCLC in Qujing had a unique driver gene mutation profile and significant differences in *EGFR* and *KRAS* mutation frequencies between men and women. The characteristics of gene mutations associated with patients with lung cancer in Qujing have been identified. However, the underlying molecular mechanisms of lung cancer in Qujing are complex and still not fully understood. On the contrary, a number of animal and *in vitro* studies showed that alveolar macrophages loaded with carbon particles from smoke led to an increased risk of respiratory tract infections. They also showed that the pathways involved in lung carcinogenesis induced by indoor coal-fired particles and that induced by tobacco smoke might be identical (18–20). However, no evidence directly demonstrated that abnormal driver gene profile was the cause of the high incidence of lung cancer in this region. Therefore, the molecular mechanism underlying the high incidence of lung cancer in the Qujing population remains to be further explored.

This study was based on the analysis of multi-gene mutations in a large number of patients in Qujing compared with patients from other regions in Yunnan Province. In the present study, a distinct profile of driver gene mutations was found in patients with NSCLC from the Qujing area. Except the differential distribution of *EGFR*, *ALK*, *ROS1*, and *KRAS* mutations, *EGFR* compound mutations as well as *KRAS* G12C and G12D also displayed a “Qujing”-specific spectrum in this study. Previous findings and the findings of this study suggested that the prevalence of common driver mutations in patients with NSCLC from Qujing was different from that in patients from other regions of China, but similar to that in Western populations (9, 10, 21–23). However, further studies are required to validate the findings.

The *EGFR* mutation rate in patients with lung cancer from Xuanwei is still controversial. Wei et al. reported that 57% (51/90) and 43% (73/168) of patients with lung cancer from Xuanwei and non-Xuanwei regions carried *EGFR* mutations, respectively (24). Hosgood et al. showed that the incidence of *EGFR* mutation was 35% in female patients (never smokers) with lung cancer in Xuanwei (9). The present study found that the *EGFR* mutation rate was 46.01% in patients with NSCLC from Qujing, which was lower than that reported by Wei et al., but higher than that reported by Hosgood et al. This inconsistency in results might be due to the differences in population selection. On the one hand, Qujing City includes Xuanwei and other counties in its administrative area, and therefore this study included patients who were not in Xuanwei but belonged to Qujing City. On the other hand, the patients were not selected according to specific clinical characteristics.

The most commonly known type of *EGFR* mutation is 19Del (accounting for ~45% of *EGFR* mutations), followed by L858R (accounting for ~40% of *EGFR* mutations). The remaining ~10% of *EGFR* mutations are defined as uncommon mutations, including exon 20 insertions (20ins), T790M, G719X, L861X, and S768I (25). However, the mutation frequencies of 19Del (20.52%) and L858R (24.28%) in patients with NSCLC from Qujing were lower than those reported in the literature. In addition, a higher proportion of *EGFR* G719X + L861Q (21.10%) and G719X + S768I (19.65%) mutation subtypes were found in patients with NSCLC from Qujing. Interestingly, *EGFR* compound mutations were more likely associated with epidemiological issues (living/working in the rural area, e.g., farmers). People who used to live or work in the rural areas of Qujing might have a higher chance of being exposed, for example, to coal-fired flue gas (4, 10). However, further large-scale investigations are warranted to confirm the correlation between *EGFR* compound mutations in NSCLC and environmental exposures in this region.

NSCLC with the coexistence of multiple *EGFR* mutations may have a unique oncogenic mechanism that may reflect the efficacy of EGFR-specific tyrosine kinase inhibitors. ERBB2 phosphorylation was markedly reduced in cells expressing L861Q plus G719X compared with lung cancer cells expressing L861Q alone. The viability assays revealed that lung cancer cells expressing L861Q + G719A showed decreased sensitivity (8- to 58-fold reduction) to EGFR-specific inhibitors, erlotinib and osimertinib, compared with cells expressing L861Q alone, but pan-ERBB inhibitors exerted superior growth-inhibitory effects on cells expressing compound L861Q/G719X mutations (26). Similarly, the cells co-expressing G719X and S768I also showed a good response to afatinib, a pan-ERBB inhibitor (27). In this study, a higher proportion of *EGFR* compound mutations were detected in patients with NSCLC from Qujing. Therefore, pan-ERBB inhibitors exerted superior tumor-growth-inhibitory effects in these patients compared with EGFR-specific inhibitors. Further clinical data should be collected to confirm these cell research based findings.

*KRAS* is the second most common driver gene in lung cancer, and the frequency of *KRAS* mutation is lower in Chinese patients than in Western populations. However, the mutation frequency of *KRAS* in Qujing (including Xuanwei) was inconsistent (6.3%–29.2%) in previous reports due to the limited number of patients (9–11, 28, 29). In this study based on a large number of patients with NSCLC, the frequency of *KRAS* mutation was significantly higher in patients with NSCLC from Qujing than in those from non-Qujing regions (23.02% vs 7.85%). Targeting KRAS protein has been one of the toughest challenges in cancer treatment research. A specific mutation known as *KRAS* G12C is a major driver of tumor growth, occurring broadly across solid-tumor indications. *KRAS* G12C mutation is found in about 13% of patients with NSCLC in the United States (30), and approximately 32.3% of patients with NSCLC in China (31). In this study, *KRAS* G12C was also the main mutant subtype of *KRAS* in patients with NSCLC from Qujing (51.11%). With the development of drugs inhibiting *KRAS* G12C, this study suggested that patients with *KRAS* G12C mutations in Qujing might benefit from targeted therapy, such as AMG510 (32).

In general, previous studies based on patients with lung cancer from Xuanwei/Qujing showed that a higher proportion of *EGFR* compound mutations and *KRAS* mutations were observed, although *EGFR* mutation rate in patients with lung cancer patients from Xuanwei was still controversial (9–12, 24, 28, 29, 33–35) (**Table 4**). The cause of these specific genetic changes remains unclear. However, the main findings were as follows: people using smoky coal had an up to 30-fold higher risk of lung cancer compared with those using smokeless coal and wood (4); lung cancer patients with coal exposure history in Xuanwei had a higher *KRAS* mutation rate (11, 33) (**Table 4**); and the occupation of patients (living/working in the rural area, e.g., farmers) was independently associated with an increased rate of *EGFR* compound mutations in this study. These findings suggested that environmental exposure might be an important reason for the specific mutation spectrum in this area. Hence, further large-scale investigations are warranted to confirm the correlation between the driver gene profile of patients with lung cancer and the environmental exposure in this region.

**Table 4:** *EGFR* and *KRAS* mutation characteristics in patients with lung cancer from Xuanwei/Qujing in previous studies.

Study	Gene	Patients	n	Mutation rate
Hosgood 3rd, et al. (9)	EGFR	NSCLC, female, non-smoking	40	35%
	KRAS	NSCLC, female non-smoking	40	15%
Ma et al. (34)	EGFR	NSCLC	119	EGFR: 39.45% G719X+S768I: 22.69% G719X+L861Q: 0.8%
	KRAS	NSCLC	119	23.53%
DeMarini et al. (29)	KRAS	Lung tumors, female, non-smoking, smoky exposure,	24	29.2%
Keohavong et al. (10)	KRAS	No evidence of lung cancer from XuanWei County	92	2.2%
Keohavong et al. (11)	KRAS	Lung cancer	41	22.9%
Yu et al. (33)	EGFR	NSCLC, smoky coal exposure	79	37.97%
	KRAS	NSCLC, smoky coal exposure	79	29.11%
Chen et al. (24)	EGFR	NSCLC patients	90	EGFR: 57% G719X+S768I: 25.56% G719X+L861Q: 1.0%
Yang et al. (28)	EGFR	NSCLC patients	63	55.6% G719X+S768I: 17.1%
	KRAS	NSCLC patients	63	6.3%
Zhou et al. (12)	EGFR	NSCLC patients	447	EGFR: 34.9% G719X+S768I: 4.5% G719X+L861Q: 0.6%
Zhou et al. (35)	EGFR	NSCLC patients	212	EGFR: 30.1%

On the other hand, some studies showed that patients with *EGFR* mutations in Xuanwei had a poor prognosis after receiving EGFR-TKI treatment. This might be due to the high incidence of rare *EGFR* mutations in this area (12, 36). These studies also found that the incidence of uncommon *EGFR* mutations and *EGFR* compound mutations was high in patients with NSCLC from Qujing. Therefore, the information on the significance of these mutations in targeted treatment deserves further investigation due to the high incidence of NSCLC with the so-called uncommon *EGFR* mutations in the Qujing population. On the contrary, chemotherapy is usually less effective in patients with NSCLC having *KRAS* mutations (37). Many novel treatment strategies have been developed, including targeting downstream signaling pathways (38), directly targeting KRAS (39), and using immunotherapy (40). Of these, immunotherapy may be one of the most promising treatment strategies for patients with NSCLC having *KRAS* mutations. Thus, we hope that these treatment strategies will bring clinical benefits to patients with lung cancer having *KRAS* mutations in Qujing in the future.

The incidence of lung cancer in the Qujing City of China is very high, and the related mortality is also high. Therefore, a comprehensive understanding of the molecular characteristics of patients with lung cancer in this region may provide the basis for a precise diagnosis and treatment. A major strength of this study was the large number of patients with NSCLC included to estimate the prevalence of common actionable genomic alterations (involving *EGFR*, *ALK*, *ROS1*, *KRAS*, *BRAF*, *RET*, *MET*, *HER2*, *NRAS*, and *PIK3CA*) in Qujing. These estimates can serve as a reference for future research. However, this study also had several limitations. First, it was a retrospective analysis and included only a single institution. Second, not all patients underwent the same molecular testing. Furthermore, the data on the treatment and prognosis of these patients were not collected, and therefore whether these patients could benefit from targeted therapy was unclear.

## Conclusion

In conclusion, this study displayed a unique profile of driver gene mutations in patients with NSCLC in Qujing. More patients with NSCLC in Qujing harbored *EGFR* G719X + S768I and *EGFR* G719X + L861Q compound mutations, besides 19DEL and L858R. Also, patients with NSCLC in Qujing had a higher proportion of *KRAS* (G12C) mutations. Therefore, these findings suggested that different treatment strategies should be adopted in patients with NSCLC in Qujing.

## Data Availability Statement

The datasets presented in this study can be found in online repositories. The names of the repository/repositories and accession number(s) can be found in the article/**Supplementary Material**.

## Ethics Statement

This study was conducted with approval from the Institutional Review Board of Yunnan Cancer Hospital. Informed consent was waived because of the retrospective nature of this study, and the de-sensitized clinical data were collected.

## Author Contributions

YH, YZ, and HS conceived and designed the experiments. FG, YD, QL, JC, XL, YG, ZS, and LD collected the data. YZ, ZH, FY, and CZ analyzed the data and wrote the manuscript. All authors read and approved the final manuscript.

## Funding

This study was partially supported by The National Natural Science Fund (No. 81860513, and No. 81960335).

## Conflict of Interest

ZH, FY, and CZ were employed by Amoy Diagnostics Co., Ltd.

The remaining authors declare that the research was conducted in the absence of any commercial or financial relationships that could be construed as a potential conflict of interest.

## Supplementary Material

Supplementary Figure 1 Mutation frequencies of *EGFR*, *ALK*, *ROS1*, and *KRAS* in patients with lung adenocarcinoma from Qujing and non-Qujing areas.

Supplementary Figure 2 Co-mutations of driver genes in patients with NSCLC from Qujing and non-Qujing areas.

## Abbreviation

EGFR, Epidermal growth factor receptor; ALK, Anaplastic lymphoma kinase; ROS1, Proto-oncogene receptor tyrosine kinase; KRAS, Kirsten rat sarcoma 2 viral oncogene homolog; BRAF, V-raf murine sarcoma viral oncogene homolog B; RET, Ret Proto-Oncogene; MET, MET proto-oncogene receptor tyrosine kinase; HER2, Epidermal growth factor receptor 2; NRAS, Neuroblastoma RAS viral oncogene homolog; PIK3CA, Phosphatidylinositol-4,5-Bisphosphate 3-Kinase Catalytic Subunit Alpha.
