# Supplementary figures and images for: Unique Profile of Driver Gene Mutations in Patients With Non-Small-Cell Lung Cancer in Qujing City, Yunnan Province, Southwest China
Source: Front Oncol. 2021 Apr 13;11:644895. doi: 10.3389/fonc.2021.644895 (PMC8076749; doi:10.3389/fonc.2021.644895)

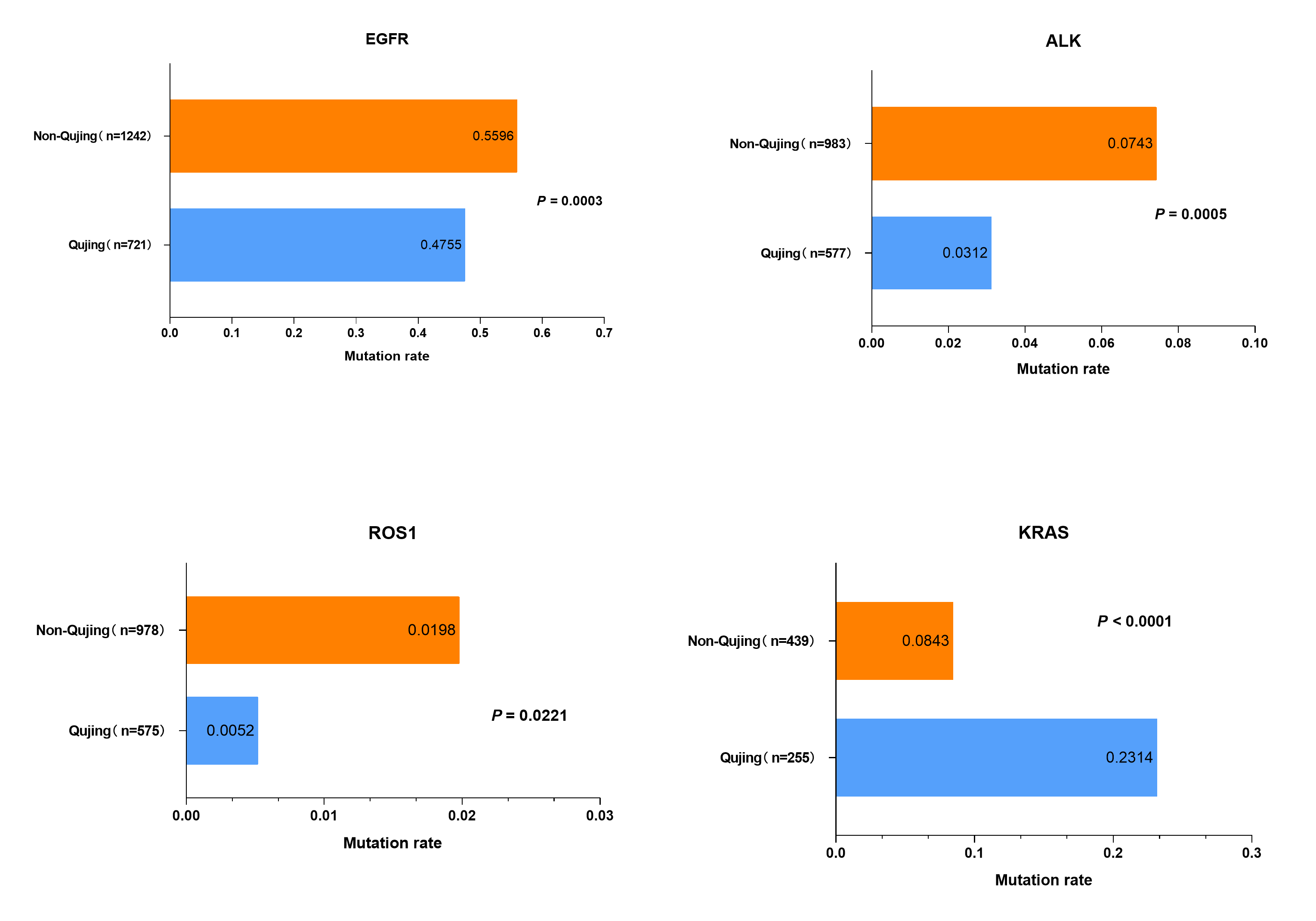

Supplement: Supplementary Figure 1 — Mutation frequencies of EGFR, ALK, ROS1, and KRAS in patients with lung adenocarcinoma from Qujing and non-Qujing areas. [file Image_1.tif]

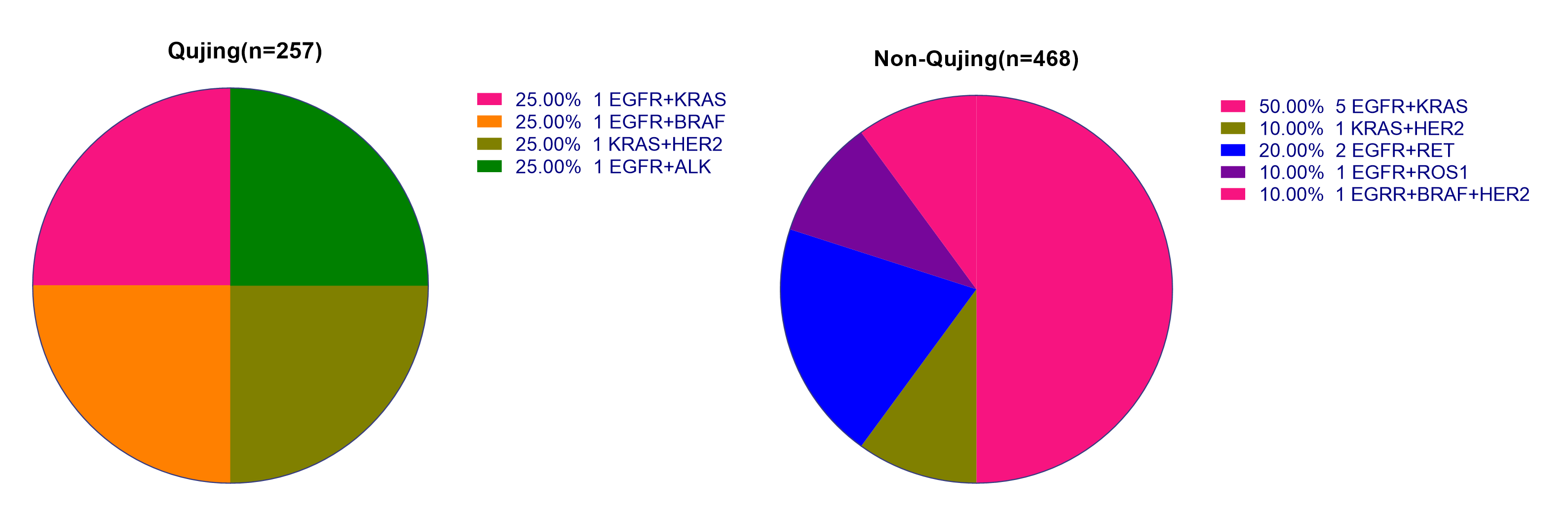

Supplement: Supplementary Figure 2 — Co-mutations of driver genes in patients with NSCLC from Qujing and non-Qujing areas. [file Image_2.tif]
