# Supplementary material for: Unique Profile of Driver Gene Mutations in Patients With Non-Small-Cell Lung Cancer in Qujing City, Yunnan Province, Southwest China
Source: Front Oncol. 2021 Apr 13;11:644895. doi: 10.3389/fonc.2021.644895 (PMC8076749; doi:10.3389/fonc.2021.644895)
Supplement: Supplementary file 3 [file Table_1.docx]

**Supplementary Table 1. Experimental methods for the detection of *EGFR, ALK, ROS1, KRAS, BRAF, RET, MET, HER2, NRAS,* and *PIK3CA* in detail**

| **Method** | **Kit** | **Company/Institution** | **Gene** | **Number** |
| --- | --- | --- | --- | --- |
| ARMS-PCR | EGFR Mutations Detection Kit® | Amoy Diagnostics | EGFR | 436 |
| ARMS-PCR | BRAF Mutations Detection Kit ® | Amoy Diagnostics | BRAF | 3 |
| ARMS-PCR | HER2 Mutations Detection Kit ® | Amoy Diagnostics | HER2 | 2 |
| ARMS-PCR | MET Mutations Detection Kit ® | Amoy Diagnostics | MET | 6 |
| ARMS-PCR | EGFR Mutations Detection Kit ®, ALK Fusion Gene Detection Kit ® | Amoy Diagnostics | EGFR/ALK | 6 |
| ARMS-PCR | EGFR Mutations Detection Kit ®, BRAF Mutations Detection Kit ® | Amoy Diagnostics | EGFR/BRAF | 1 |
| ARMS-PCR | EGFR Mutations Detection Kit ®, KRAS Mutations Detection Kit ® | Amoy Diagnostics | EGFR/KRAS | 3 |
| ARMS-PCR | EGFR Mutations Detection Kit ®, NRAS Mutations Detection Kit ® | Amoy Diagnostics | EGFR/NRAS | 1 |
| ARMS-PCR | ALK/ROS1 Fusion Gene Detection Kit ® | Amoy Diagnostics | ALK/ROS1 | 2 |
| ARMS-PCR | EGFR Mutations Detection Kit ®, ALK/ROS1 Fusion Gene Detection Kit ® | Amoy Diagnostics | EGFR/ALK/ROS1 | 859 |
| ARMS-PCR | EGFR Mutations Detection Kit ®, ALK Fusion Gene Detection Kit ®, KRAS Mutations Detection Kit ® | Amoy Diagnostics | EGFR/ALK/KRAS | 1 |
| ARMS-PCR | KRAS Mutations Detection Kit ®, ALK/ROS1 Fusion Gene Detection Kit ® | Amoy Diagnostics | KRAS/ALK/ROS1 | 1 |
| ARMS-PCR | KRAS Mutations Detection Kit ®, NRAS Mutations Detection Kit ®, BRAF Mutations Detection Kit ® | Amoy Diagnostics | KRAS/NRAS/BRAF | 1 |
| ARMS-PCR | EGFR/ALK/ROS1 Mutation Detection Kit ®, MET Mutations Detection Kit ® | Amoy Diagnostics | EGFR/ALK/ROS1/MET | 76 |
| ARMS-PCR | EGFR/ALK/ROS1 Mutation Detection Kit ®, KRAS Mutation Detection Kit ® | Amoy Diagnostics | EGFR/ALK/ROS1/KRAS | 15 |
| ARMS-PCR | EGFR/ALK/ROS1 Mutation Detection Kit ®, NRAS Mutation Detection Kit ® | Amoy Diagnostics | EGFR/ALK/ROS1/NRAS | 4 |
| ARMS-PCR | EGFR/ALK/ROS1 Mutation Detection Kit ®, BRAF Mutation Detection Kit ® | Amoy Diagnostics | EGFR/ALK/ROS1/BRAF | 1 |
| ARMS-PCR | EGFR/ALK/ROS1 Mutation Detection Kit ®, BRAF Mutation Detection Kit ® | Amoy Diagnostics | EGFR/ALK/ROS1/BRAF | 1 |
| ARMS-PCR | EGFR/ALK/ROS1 Mutation Detection Kit ®, KRAS Mutation Detection Kit ®, NRAS Mutation Detection Kit ® | Amoy Diagnostics | EGFR/ALK/ROS1/KRAS/NRAS | 1 |
| ARMS-PCR | EGFR/ALK/ROS1 Mutation Detection Kit ®, KRAS Mutation Detection Kit ®, MET Mutation Detection Kit ® | Amoy Diagnostics | EGFR/ALK/ROS1/KRAS/MET | 1 |
| ARMS-PCR | EGFR/ALK/ROS1/KRAS/NRAS/BRAF/PIK3CA/HER2/RET/MET Mutation Detection Kit ® | Amoy Diagnostics | EGFR/ALK/ROS1/KRAS/NRAS/BRAF/PIK3CA/HER2/RET/MET | 725 |
| NGS | EGFR/ALK/ROS1/KRAS/BRAF/HER2/RET/MET | Burning Rock Dx | EGFR/ALK/ROS1/KRAS/BRAF/HER2/RET/MET | 205 |
| NGS | EGFR/ALK/ROS1/KRAS/NRAS/BRAF/PIK3CA/HER4/RET/MET | Amoy Diagnostics | EGFR/ALK/ROS1/KRAS/NRAS/BRAF/PIK3CA/HER4/RET/MET | 221 |
| NGS | Multi-gene NGS Panel | Yunnan Cancer Hospital/LDT | Multi-gene NGS Panel（≥EGFR/ALK/ROS1/KRAS/NRAS/BRAF/PIK3CA/HER4/RET/MET） | 100 |
| Total | | | | 2672 |

**Supplementary Table 2. Characteristics of 526 patients with NSCLC tested using NGS from Qujing and non-Qujing areas**

| **Characteristics** | **All patients** | **Region** | | ***P*-value** |
| --- | --- | --- | --- | --- |
|  | **(n = 526)** | **Qujing (n = 188)** | **Non-Qujing(n = 338)** |  |
| **Gender** |  |  |  |  |
| Male | 272 | 104 | 168 |  |
| Female | 254 | 84 | 170 | 0.2168 |
| **Age** |  |  |  |  |
| Median (range) |  |  |  |  |
| ≤ 40 | 25 | 6 | 19 |  |
| > 40 | 501 | 182 | 319 | 0.2094 |
| **Histopathology** |  |  |  |  |
| Adenocarcinoma | 485 | 182 | 303 |  |
| Squamous  carcinoma/Unknown | 41 | 6 | 35 | 0.0033 |
| Unknown (NSCLC) |  |  |  |  |
| **Smoking history** |  |  |  |  |
| Yes | 163 | 67 | 96 |  |
| No/Unknown | 363 | 121 | 242 | 0.0855 |
| Unknown | 4 | 2 | 2 |  |
| **Family history** |  |  |  |  |
| Yes | 57 | 26 | 31 |  |
| No | 469 | 162 | 307 | 0.0955 |
| **Staging** |  |  |  |  |
| I-Ⅲa | 229 | 98 | 131 |  |
| Ⅲb-Ⅳ | 199 | 53 | 146 |  |
| Unknown | 98 | 37 | 61 | 0.1924 |
| **Lesion site** |  |  |  |  |
| Left | 204 | 82 | 122 |  |
| Right | 311 | 106 | 205 |  |
| Unknown | 11 | 0 | 11 | 0.4829 |
| **Occupation** |  |  |  |  |
| Farmer | 212 | 100 | 112 |  |
| Non-farmer/Unknown | 314 | 88 | 226 | < 0.0001 |

**Supplementary Table 3. Mutation frequencies of lung cancer driver genes in patients with NSCLC tested using NGS from Qujing and non-Qujing areas**

| **Gene** | **Total** | **Qujing** | | | | **Non-Qujing** | | | | ***P*-value** |
| --- | --- | --- | --- | --- | --- | --- | --- | --- | --- | --- |
|  |  | **Total** | **WT** | **MUT** | **Rate** | **Total** | **WT** | **MUT** | **Rate** |  |
| EGFR | 526 | 188 | 102 | 86 | 45.74% | 338 | 192 | 146 | 43.20% | 0.5725 |
| ALK | 526 | 188 | 185 | 3 | 1.60% | 338 | 317 | 21 | 6.21% | 0.0154 |
| ROS1 | 526 | 188 | 188 | 0 | 0.00% | 338 | 333 | 5 | 1.48% | n/a |
| KRAS | 526 | 188 | 143 | 45 | 23.94% | 338 | 310 | 28 | 8.28% | < 0.0001 |
| BRAF | 526 | 188 | 184 | 4 | 2.13% | 338 | 331 | 7 | 2.07% | 0.9999 |
| HER2 | 526 | 188 | 184 | 4 | 2.13% | 338 | 326 | 12 | 3.55% | 0.4376 |
| RET | 526 | 188 | 186 | 2 | 1.06% | 338 | 332 | 6 | 1.78% | 0.7177 |
| MET | 526 | 188 | 187 | 1 | 0.53% | 338 | 337 | 1 | 0.30% | 0.9999 |
| NRAS | 320 | 105 | 105 | 0 | 0.00% | 215 | 215 | 0 | 0.00% | n/a |
| PIK3CA | 320 | 105 | 105 | 0 | 0.00% | 215 | 209 | 6 | 2.79% | n/a |

n/a: not applicable, WT: wild type; MUT: mutation type

**Supplementary Table 4. *KRAS* mutation status in patients with NSCLC from Qujing and non-Qujing areas with or without a smoking history**

|  |  | **KRAS** | | | |  |
| --- | --- | --- | --- | --- | --- | --- |
| **Qujing and Non-Qujing** | Total | Mutation | WT | Mutation Rate | | *P*-value |
| Somking | 208 | 41 | 167 | 19.70% | | **0.001** |
| Non-Somking | 536 | 58 | 477 | 10.80% | |  |
|  |  | **KRAS** | | | |  |
| **Somking** | Total | Mutation | WT | | Mutation Rate | *P*-value |
| Qujing | 77 | 26 | 51 | | 35.10% | **< 0.0001** |
| Non-Qujing | 131 | 15 | 116 | | 11.50% |  |
|  |  | **KRAS** | | | |  |
| **Non-Somking** | Total | Mutation | WT | | Mutation Rate | *P*-value |
| Qujing | 187 | 35 | 152 | | 18.70% | **< 0.0001** |
| Non-Qujing | 349 | 23 | 325 | | 6.60% |  |
|  |  | **KRAS** | | | |  |
| **Non-Qujing** | Total | Mutation | WT | | Mutation Rate | *P*-value |
| Somking | 131 | 15 | 116 | | 11.50% | 0.081 |
| Non-Somking | 349 | 23 | 325 | | 6.60% |  |
|  |  | **KRAS mut and WT** | | | |  |
|  | Total | Somking | Non-Somking | | Somking Rate | *P*-value |
| Qujing | 264 | 77 | 187 | | 29.20% | 0.586 |
| Non-Qujing | 480 | 131 | 349 | | 27.30% |  |
